# I Like It Here: A Life in Filmmaking

**DOI:** 10.1093/geroni/igaf122.1680

**Published:** 2025-12-31

**Authors:** Ralph Arlyck

**Affiliations:** Columbia Journalism, Germantown, New York, United States

## Abstract

Ralph Arlyck, age 85, has been making acclaimed and award-winning movies since the mid-60s, when he was a graduate student at San Francisco State. His most recent film, “I Like It Here” explores death and the fear of being left alone. Ralph wanders through his neighborhood and his own past to understand what’s happening within his body and brain — and those of the friends he encounters along the way. Topics swing from the mundane to the existential, seeking answers to complex questions, ie what it’s like to feel your joints and thoughts stiffen but to be more relaxed about getting ahead or keeping up. The film’s title has a double meaning: “here” refers to both the filmmaker’s green, rural surroundings and the preciousness of life itself. Ralph will share footage from the film and discuss his perspective on what it means to be an older artist actively engaged in a arts practice that has lasted over 60 years.

